# Nutrition in Children’s Growth and Development

**DOI:** 10.3390/nu18142356

**Published:** 2026-07-17

**Authors:** Cristina Oana Mărginean, Maria Oana Săsăran

**Affiliations:** 1 Department of Pediatrics 1, “George Emil Palade” University of Medicine, Pharmacy, Science and Technology of Targu Mures, Georghe Marinescu Street No 38, 540136 Targu Mures, Romania; 2 Department of Pediatrics 3, “George Emil Palade” University of Medicine, Pharmacy, Science and Technology of Targu Mures, Georghe Marinescu Street No 38, 540136 Targu Mures, Romania; oanam93@yahoo.com

It is an honor and a privilege to have helped edit and bring this Special Issue titled “Nutrition in Children’s Growth and Development” to you.

Children’s growth and nutrition involve complex biological processes; the former requires adequate nutrient intake in order to reduce the incidence of malnutrition in vulnerable areas, with impacts on neuro-psychomotor development. Nutrient and vitamin deficiencies may cause isolated symptoms, silent evolvement and various serious dysfunctions such as concentration problems or neuropsychiatric disorders, as well as predisposing risk factors for infections [1].

Early childhood nutritional status is greatly impacted by the mother’s pre-conceptional and pregnancy nutrition, anthropometric parameters and pregnancy-related weight gain [2]. The first 1000 days, beginning from conception towards the age of two years, is a critical period with impacts on the neurodevelopment of the child and on their lifelong health. Due to these considerations, the adequate intake of protein, iron, zinc, choline, folate, iodine, vitamins A/D/B6/B12, and long-chain polyunsaturated fatty acids during this period of life plays a big role [3]. Proteins and long-chain polyunsaturated fatty acids are related to neuronal membrane formation. Iron, zinc, choline, folate, and iodine are involved in synaptogenesis, and vitamins A, D, B6, and B12 are responsible for neurotransmitter synthesis [3].

The first five years of life constitute an extremely vulnerable period, with moderate or severe wasting being responsible for high rates of morbidity and mortality [4]. Integrated nutrition and correct interventions to ensure a healthy nutrition can ensure a strong developmental status for each child [5]. On the other hand, inappropriate, imbalanced nutrition and excessive caloric intake can lead to obesity, a pandemic disease at pediatric ages whose adulthood-related complications originate in adolescence [6].

Dietary changes reflect on the gut microbiome and its variability, which can trigger bothersome digestive symptoms, as the gut microbiota play an essential role in maintaining the integrity of the intestinal barrier [contribution 1]. The unknown relationship between nutritional status and microbiome imbalances represents a cornerstone for causal effects of the gut microbiome on gastrointestinal disorders [contribution 1]. Ultimately, nutrition is responsible for a child’s health and for ensuring their normal growth and development.

The aim of this Special Issue is to showcase reviews and research articles from pediatricians, obstetricians, and nutritionists from all over the world, focusing on the causality between dietary patterns, maternal diet, and children’s health. Moreover, this Special Issue will also cover topics related to nutrient and vitamin deficiencies, microbiome imbalances, and their impact on children’s well-being and anthropometric parameters.

An important topic covered in this Special Issue is fetal growth restriction (FGR) and postnatal catch-up growth, which is a central adaptive process with impacts on health and metabolic risk factors. For 10 years, the authors followed up on children born at term who were prenatally affected by growth restriction, and they observed that infants with severe FGR experienced a higher early catch-up in weight and length at 1 and 2 years of age, without any differences related to gender, and with a similar BMI at the ages of 5 and 10 years to those with moderate FGR, together with a mitigated metabolic risk [7].

Maternal nutrition can have a definitory impact on the child’s nutritional status and related metabolic complications. Maternal obesity, gestational diabetes, and poor diet quality during pregnancy were correlated with liver steatosis in offspring in the study of DiStefano et al. The authors emphasized that intrauterine exposure to dyslipidemia and hyperglycemia can be related to early lipid deposition within hepatocytes. In addition, breastfeeding has a protective role against metabolic dysfunction-associated steatotic liver disease (MASLD), whereas formula feeding and processed food intake could increase the risk of later steatosis. On this note, the authors underline the importance of early-life windows for adequate dietary intervention in order to reduce the incidence of pediatric MASLD [contribution 2]. Maternal dietary practices also reflect on the child’s nutrition. Długoński et al. investigated the impact of maternal nutrition on the child’s health and underlined the importance of socio-demographic determinants of maternal feeding practices in the nutrition of school-aged children, which should be tailored to family structure and mothers’ employment status. In addition, mothers with only one child seem to enforce dietary rules and monitoring measures less often than mothers with multiple children [contribution 3].

Another important public health problem is stunting—chronic malnutrition—in vulnerable geographical areas, such as South Asia. Dietary interventions include large-scale programs such as the Central Asia Stunting Initiative (CASI), which aims to decrease the incidence and effects of stunting. Hussain et al. performed a cross-sectional baseline and midline survey as part of a pre- and post-intervention evaluation, which was applied within the households of children aged 0–59 months. CASI interventions substantially improved child nutrition and maternal behaviors but also implied sustained strategies concerning food insecurity, economic empowerment, and long-term resilience [contribution 4].

Bad dietary habits can impact anthropometry and child health. Concurring factors related to improper feeding schedules represent another hot research topic. Skipping breakfast is a common problem among adolescents. A study performed on high school students from the Korea Youth cohort between 2018 and 2024 compared more than 11,000 students who lived in dormitories with those living at home (n = 164,446). Students living in dormitories had higher rates of breakfast days than those living at home. Still, in both study groups many adolescents skipped breakfast, and the authors concluded that it is necessary to enhance awareness on the importance of the first meal of the day [contribution 5]. Moreover, Seni et al. showed that bad habits such as sugary food intake and anthropometric abnormalities such as higher body mass index (BMI) and/or waist circumference are positively correlated with dental caries in Romanian school-age children [contribution 6].

Dietary practices are also very important in children with inborn metabolic conditions such as lysosomal storage disorder. A lot of children with late-onset Pompe disease (LOPD) remain asymptomatic until later years. However, these children are predisposed to becoming overweight or obese. Thus, a high-protein, low-carbohydrate diet is highly recommended in children with LOPD. The enforcement of nutrition guidelines for children with LOPD is recommended, which could focus on providing personalized dietary intake, useful for adequate growth, and feeding/swallowing practices [contribution 7].

Different food choices impact the gut microbiota and, secondarily, mental health, because gut microbiota are involved in the synthesis of neurotransmitters such as serotonin, dopamine, and noradrenaline. Dysbiosis, associated with systemic inflammation, contributes to the onset of psychiatric disorders such as depressive disorders, anxiety, attention-deficit hyperactivity disorder (ADHD) and autism. The authors observed that a diet rich in sugar and low in fiber produces dysbiosis and is associated with poor mental health. Therefore, integrative strategies are needed to promote microbiome regulation [contribution 8].

Variations in human milk metabolite composition in women with gestational diabetes mellites (GDM) suggest that GDM could influence milk composition, with impacts on the offspring’s growth. The study of Fradet et al. observed that human milk metabolites of mothers with GDM are characterized by higher levels of myristic acid, glycerol, uracil, arachidonic acid, and cholesterol, as well as α-ketoglutarate and glycine, which are all negatively correlated with infant growth. The authors concluded that GDM can influence mammary gland biology and delivery, with effects on human milk composition [contribution 9].

Complementary feeding (CF) practices vary greatly around the world. The baby-led weaning (BLW) method implies self-feeding and early introduction of solid foods in the infant’s meals. A study conducted in Poland showed that BLW is associated more frequently with prolonged breastfeeding, as well as with tasting and accepting various food textures, as opposed to those who were exposed to traditional CF. Moreover, the authors concluded that this method made children less likely to consume milk formula and pudding-type products. BLW, despite the risk of choking, which imposes careful parental supervision, also supports motor development and healthy eating habits [contribution 10].

Vitamin D plays an important role not only in bone metabolism but also in immunity and defense against infections. Infant levels seem to be greatly influenced by maternal vitamin D status. Another study investigated the prevalence of vitamin D deficiency and its impact on the anthropometric outcomes of newborns in a group of Roma mothers and their infants, which constitute a vulnerable population; the authors observed that all mothers and almost all newborns had vitamin D insufficiency or deficiency. These findings emphasize the need for vitamin D supplementation during pregnancy for prevention of hypovitaminosis D in offspring [contribution 11].

Another study conducted on juvenile mice studied the role of folic acid (FA) and colostrum basic protein (CBP)-fortified milk powder in growth, bone development and metabolic safety following an 8-week intervention. The study enrolled three study groups (control, fortified milk and positive control group) and demonstrated that FA-CBP-fortified milk significantly enhanced linear growth during puberty, with bone mass and microstructure that become fortified in early adulthood, these effects being explained by transient activation of the GH–IGF-1 axis [contribution 12].

Through the present Special Issue, we aimed to emphasize that a multidisciplinary and transdisciplinary approach in children’s growth and nutrition is useful for providing correct management, especially in cases in which dietary imbalances need to be corrected or the presence of underlying disorders call for special nutrition patterns. The topics addressed within this article collection cover the vast majority of pediatric growth and dietary pathology and include ten research articles and two reviews. All the articles have been written by figures with high levels of expertise in their fields and have focused on the clinical management of the disorders which they specialize in. All these articles bring novelty to the literature data and enhance progress in the field of pediatric nutrition and gastroenterology. Editing this Special Issue was a wonderful and uplifting purpose for me, providing a learning opportunity, and I hope that the authors felt the same.
